# The Effect of Preparation Size on Efficacy of Smear Layer Removal; A Scanning Electron Microscopic Study

**DOI:** 10.7508/iej.2015.03.005

**Published:** 2015-07-01

**Authors:** Mehdi Tabrizizadeh, Ameneh Shareghi

**Affiliations:** a *Department of Endodontics, Dental School, Shahid Sadoughi University of Medical Sciences, Yazd, Iran; *; b *Department of Endodontics, Dental School, Shahrekord University of Medical Sciences, Shahrekord, Iran.*

**Keywords:** Dentinal Tubules, Ethylenediaminetetraacetic Acid, EDTA, Root Canal Preparation, Scanning Electron Microscopy, Smear Layer

## Abstract

**Introduction:**

Enlargement of the root canal may potentially affect efficient smear layer (SL) removal. The aim of the present *in vitro* study was to compare SL removal following canal preparation with two different sizes/tapers by means of scanning electron microscopy (SEM).

**Methods and Materials:**

A total of 50 extracted human mandibular premolars were decoronated. The teeth were randomly divided into two experimental groups (*n*=20) and two negative control groups. In groups 1 and 2 the sizes of master apical file (MAF) were #25 and 40, respectively. Coronal part of the canals were flared with #2 Piezo drills in group 1 and sizes #2 to 6 in group 2. Finally FlexMaster NiTi rotary instruments were used to complete canal preparation (25/0.04 and 35/0.06 in groups 1 and 2, respectively). The irrigation protocol consisted of 10 mL of 17% ethylenediaminetetraacetic acid (EDTA) for 1 min followed by 10 mL of 5.25% NaOCl for 3 min. The patency of dentinal tubules was evaluated under SEM with Hülsmann scores. Data were analyzed with the Kruskal-Wallis and Mann-Whitney U tests.

**Results:**

The number of patent dentinal tubules in coronal third of the group 1 was significantly more than group 2 (*P*<0.001). However, this difference was not significant for the middle and apical segments. There was a significant difference in the number of patent dentinal tubules between coronal, middle and apical thirds (*P*<0.05).

**Conclusion:**

Increasing the canal preparation size did not lead to better cleanliness of the canal walls and more efficient smear layer removal.

## Introduction

Chemomechanical or biomechanical preparation of the root canal system is one of the most important phases of endodontic treatment [1, 2]. Cleaning and shaping of the root canals not only assists in obtaining the biological objectives such as bacterial reduction, but also facilitates the three-dimensional obturation of the root canal system and placement of a high quality root filling [3]. According to Schilder [4], canals should be prepared with a continuous tapering funnel-shape manner, from the coronal to the apical ending and the apical opening should be kept as small as practical.

There is no consensus regarding the effect of apical preparation size on better removal of infected dentin or promoting the effectiveness of endodontic irrigants; some clinicians advocate smaller apical preparation size combined with tapered shapes [5]. It is clear that over-enlargement of the canals by removing more dentin form the canal walls, may lead to preparation errors such as zipping, canal transportations and perforations and also increases the risk of vertical root fracture in future [6, 7]. 

The effect of final apical preparation size (*aka* the master apical file, MAF) on treatment outcome has been evaluated in two long-term studies. Kerekes and Tronstad [8] reported similar prognosis after apical preparation to ISO sizes 20 to 40 and 45 to 100. Whereas Strindberg [9] found a poorer prognosis for larger apical preparation. Also, the results of a Toronto study [10] on endodontic outcomes favored smaller preparations in comparison with larger apical shapes (90% and 80% success rates, respectively). A randomized controlled clinical trial evaluated the effect of different MAF sizes on the outcome of primary endodontic treatment [11]. According to this study, the enlargement of the canal to 3 sizes larger than FABF (first apical binding file) is adequate, and further enlargement does not provide any additional benefit during root canal therapy.

**Figure 1:**
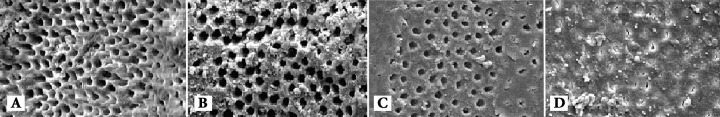
Hülsmann scoring system; *A)* *score 1*; no SL, patent dentinal tubules, *B) score2*; small amount of SL and open dentinal tubules in more than 50% of the surfaces, *C) score 3*; homogenous SL along almost the entire canal walls with less than 50% open dentinal tubules and *D) score 4*; the entire root canal walls covered with a homogenous SL and no patent dentinal tubules

Major parameters of root canal cleanliness after endodontic treatment has been evaluated using longitudinal and horizontal sections of extracted teeth [12, 13]. Smear layer (SL) is a superficial mud like layer consisting of inorganic debris and organic materials like pulp tissue, odontoblastic processes, necrotic debris, microorganisms and their metabolic byproducts which are produced during instrumentation of the canal walls [14].

The question of keeping or removing the SL remains controversial and conflicting. Reports exist regarding the removal of SL before root canal filling [15-20]. Although some researchers advocate the maintenance of SL because it occludes the patent dentinal tubules and entombs the microorganisms in the tubules [21], a systematic review and meta-analysis revealed that overall consensus has moved towards favoring the removal of the SL [22]. Different methods, irrigating solutions and chelating agents have been used to remove the SL [23]. Currently, the subsequent use of 17% ethylenediaminetetraacetic acid (EDTA) and sodium hypochlorite (NaOCl) is the recommended regiment for removal of the inorganic and organic components of the SL, respectively [24]. 

In a recent study, Tabrizizadeh *et al.* [25] showed that the amounts of microleakage through root canal fillings are directly related to the size and taper of root canal preparation and reducing the preparation size may lead to less microleakage.

Therefore, the purpose of the present study was to investigate the influence of MAF size on root canal cleanliness by observation of the presence of SL in the coronal, middle and apical thirds using scanning electron microscopy (SEM).

## Materials and Methods

For this *in vitro *study, 50 extracted human mandibular premolars with single straight roots were selected and stored in 10% formalin. All teeth had closed apices without any cracks or severe curvatures. All calculus and soft tissue remnants were removed from the root surfaces and then teeth were decoronated 14 mm from the apex.

After removal of the pulp tissue from the canals using a barbed broach, the working length (WL) was determined by inserting a #15 K-file (Dentsply Maillefer, Ballaigues, Switzerland) into the root canal until the file tip became visible from the apical foramen. The root canals were prepared 1 mm short of this length. Only those canals that were navigated with a #15 file were included in the experiment. Canal preparation in all samples were done by one person. The teeth were then randomly divided into 2 experimental and 2 control groups. 

In group 1 (*n*=20) the canals were prepared with balanced force technique using K-files with MAF size kept at #25. After every instrumentation, canals were rinsed with 2 mL of 5.25% NaOCl delivered with a 30-gauge needle (Supa, Tehran, Iran) which was inserted 1-2 mm short from the WL without any engagement with root canal walls. The coronal portion of the canals were then flared with #2 Piezo drills (Mani, Tochigi, Japan). Flaring was followed by irrigation with 2 mL of 2.5% NaOCl. Then the instrumentation was completed with FlexMaster rotary files (VDW, Munich, Germany) sizes 20/0.02, 20/0.04 and then 25/0.04 installed on a gear reduction handpiece connected to an electric torque-controlled motor (Endo-Mate TC, NSK, Nakanishi Inc., Tokyo, Japan) with torque and speed of 1.5 Ncm and 400 rpm, respectively [25].

In group 2 (*n*=20) the apical preparation size was set at #40. Then Piezo drills #2 to 6 were used for flaring the coronal part of the canals so that each successively larger drill penetrated 1 to 2 mm deeper than the previous size. Piezo drill #6 (1.7 mm diameter) was only inserted to the depth of the cutting flutes. Then rotary instrumentation was performed with FlexMaster files 35/0.02, 40/0.02 and then 40/0.04. Canal irrigation was done similar to group 1.

After canal preparation in both groups, each canal received a final irrigation with 10 mL of 17% EDTA (pH=7.7) (i-dental, Siauliai, Lithuania) for 1 min followed by 10 mL of 5.25% NaOCl for 3 min, to remove the SL [26]. Subsequently, canals were irrigated with 10 mL of normal saline and dried with paper points. For 5 teeth in negative control group the SL was not removed. 

**Figure 2:**
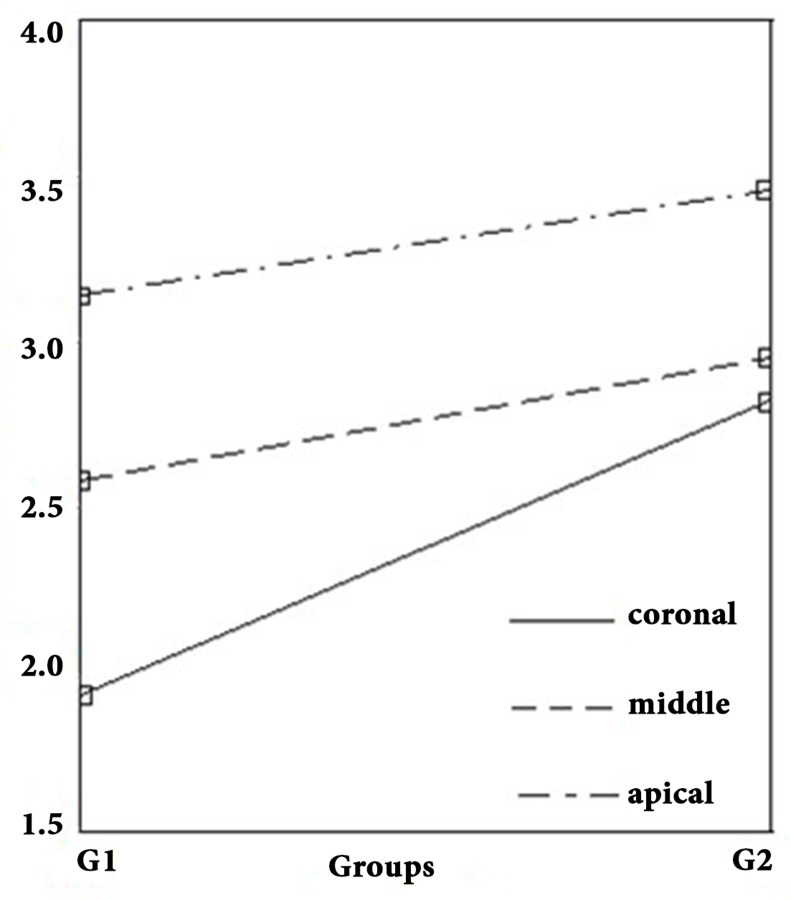
Mean Hulsmann scores in different regions of canals in two experimental groups

A longitudinal groove by a diamond disk (Axis, Sybron Endo, Sybron Dental, Anaheim, CA, USA) was created in the buccal and lingual surfaces and then the roots were split with a chisel. One half of each tooth was randomly selected to be evaluated by SEM (SEM-LEO 440, Leo Electron Microscopy Ltd, Cambridge, UK). The samples were dried in ethanol solution and coated with 10% gold-palladium for this purpose. Then photomicrographs of 2500× magnification in the coronal, middle and apical thirds, were taken. Selected samples were blind coded after scanning.

Two examiners scored the patency of dentinal tubules in coronal, middle and apical thirds, according to Hülsmann scoring system [12]: *score 1*; no SL and patent dentinal tubules, *score 2*; small amount of SL and open dentinal tubules in more than 50% of the surfaces, *score 3*; homogenous SL along almost the entire canal walls with less than 50% open dentinal tubules and *score 4*; the entire root canal walls covered with a homogenous SL and no patent dentinal tubules. Data was analyzed with the nonparametric Kruskal-Wallis and Mann-Whitney U tests. The level of significance was set at 0.05.

## Results

The mean values of Hülsmann scores in coronal, middle and apical regions of group 1 were 1.95, 2 and 3.15 and in group 2 the values were 2.85, 3 and 3.5, respectively. Higher Hülsmann score, demonstrates less patent dentinal tubules (Figures 1). This difference was statistically significant in coronal third (*P*<0.001), whereas no statistically significant differences was found in the middle and apical thirds (*P*<0.5) (Table 1 and Figure 2). 

In each experimental group, statistically significant differences were found between the number of patent dentinal tubules in the coronal, middle and apical regions (*P*<0.001 for group 1 and *P*=0.048 for group 2). Hülsmann score in the coronal, middle and apical regions of the negative control samples were recorded as 4.

## Discussion

The aim of the present study was to evaluate the influence of canal preparation size on the root canal cleanliness. To achieve this goal, all the prepared canals were standardized regarding the SL removal protocol but the canal preparation and MAF sizes were different.

It is difficult to estimate the effect of various pre- and intra-operative variables on the amount of the produced SL because of the considerable variation in the design of the studies [27]. In the present study, single-canal straight roots with partly equalize initial anatomy were selected. Root lengths were assimilated to be ~14 mm in all samples. Root canal treatment procedures alter the canal surface depending upon the canal anatomy, the type and sequence of used instruments and the chemicals used to facilitate debridement [28]. 

A review article introduced the effective factors on shaping outcomes with rotary NiTi files as root canal anatomy, instrument tip and its design, operator’s experience, rotation speed and specific instrument sequence [29].

According to previous studies, NiTi rotary instruments increase the number of occluded tubules in apical part due to dislodging of debris into the apical region [21]. In other studies, narrower anatomy of the apical region compared to other canal regions and less accessibility of irrigants have been suggested to be the reason for more remnants of SL in this zone [30].

In the present study, the mean Hülsmann score in different canal regions, were more in group 2 (large size and taper). This is probably due to the higher amounts of SL in this group, higher number of instruments and larger instrument sizes. Indeed with increasing the preparation size, more SL was created but the differences were significant only in the coronal region. 

Proper enlargement of the canal for transmission of irrigants such as NaOCl to the apical portion of the canal is another considerable factor in root canal preparation. Baker* et al.* [31] emphasized on the importance of irrigant volume and its potential influence on remaining debris on the root canal walls. It has been shown that the use of EDTA followed by NaOCl can produce clean dentinal surfaces that are devoid of debris [32]. So the necessity of instrumenting all dentin surfaces in the apical region of root canal is questionable [33]. If sufficient volume of irrigants reach apical areas, more conservative apical preparations may be sufficient meaning that confining the apical instrumentation zone to the least size that permits irrigant penetration 1-2 mm shorter than the WL, may be sufficient.

**Table 1:** Mean (SD), of patent dentinal tubules in different regions of the canals in two experimental groups

**Canal region**	**Group 1**	**Group 2**	***P*** **-value**
	**Mean (SD)**	**Mean (SD)**	
**Coronal**	1.95 (0.60)	2.85 (0.93)	0.001
**Middle**	2.6 (0.68)	3 (0.85)	0.156
**Apical **	3.15 (0.58)	2.85 (0.60)	0.061
**Total**	2.56 (0.7)	3.11 (0.84)	

The diameter of the canal in apical 2 mm of small canals is estimated to be equivalent to a #25 file (0.33 mm); so a 30-guage needle with the diameter of 0.31 mm can easily reach this area [29]. In the present study, despite the different number of instruments used for canal preparation in each group, the total volume of the used irrigation solution was similar in both groups (24 mL for each sample). Nevertheless, the volume of irrigating solution that is in contact with the canal walls (effective solution for rinsing) at similar time span is different depending on the canal size and it can be calculated with surface measurement in each section of canal.

For SL removal according to the standard protocol [26], canal rinsing with EDTA was followed by NaOCl rinse, as EDTA may leave the organic components of SL untouched; moreover, NaOCl can also neutralizes any remaining EDTA [34]. According to Moodnik *et al.* [35], NaOCl is beneficial in debris removal in the middle and coronal thirds but it cannot detach SL and smear plugs inside the dentinal tubules. Likewise, coronal and then middle thirds of the canals in two groups were the most clean parts in our study. 

Another effective factor mentioned in the studies, is the irrigant type and mode of usage that is highly variable. The frequency of using one file before discarding, is probably effective on the amount of produced SL and its removal which is also variable in different studies. This issue has not been considered in some studies. In the current study each file was discarded after preparation of 5 canals.

Although according to some microbiologic studies, larger apical preparation sizes may lead to a greater reduction in remaining bacteria [2], based on the *in vitro* study by Akhlaghi *et al.* [36], there were no significant differences regarding the reduction of intracanal bacteria between the teeth treated with different apical sizes/tapers. The impact of final canal shape and size on root strength and iatrogenic accidents such as apical transportation, ledge formation and file fracture should also be considered. Buchanan [37] recommends minimal apical preparation sizes (#20 or 25). Salzgeber and Brilliant [38] showed that instrumentation beyond #35 may allow pushing the irrigant beyond the apex and into the periapical tissues.

Another considerable issue in deciding about the size and tapering of the canal preparation is its impact on final canal seal and success rate of root canal treatment [11]. The main aim in root canal therapy is to save tooth and its desirable function. To achieve this goal, is not essential nor possible to sterilize the canal and make it free of microorganisms [11]. Other previous studies have shown that in optimal conditions, some of the canal surfaces remain intact and uncleaned [39]. 

According to a randomized controlled clinical trial, enlargement of the canal 3 sizes larger than the FABF, is adequate and further enlargement does not provide any additional benefit during endodontic treatment [11]. Strindburg [9] and Hoskinson *et al. *[40] suggested that an increase in the apical preparation size decreases the success rate due to endodontic accidents.

## Conclusion

According to the findings of the present study increasing the size of canal preparation does not lead to greater cleanliness and smear layer removal.
